# The Effects of Acute Neonatal Pain on Expression of Corticotropin-Releasing Hormone and Juvenile Anxiety in a Rodent Model

**DOI:** 10.1523/ENEURO.0162-19.2019

**Published:** 2019-11-14

**Authors:** Jared T. Zuke, Makaela Rice, Jacob Rudlong, Taylor Paquin, Erica Russo, Michael A. Burman

**Affiliations:** 1 Department of Psychology, University of New England; 2 Department of Biology, University of New England; 3 Center for Excellence in the Neurosciences, University of New England, 11 Hills Beach Rd. Biddeford, ME 04005

**Keywords:** amygdala, anxiety, CRF, neonatal, pain

## Abstract

Premature infants in the neonatal intensive care unit (NICU) may be subjected to numerous painful procedures without analgesics. One necessary, though acutely painful, procedure is the use of heel lances to monitor blood composition. The current study examined the acute effects of neonatal pain on maternal behavior as well as amygdalar and hypothalamic activation, and the long-term effects of neonatal pain on later-life anxiety-like behavior, using a rodent model. Neonatal manipulations consisted of either painful needle pricks or non-painful tactile stimulation in subjects’ left plantar paw surface which occurred four times daily during the first week of life [postnatal day (PND)1–PND7]. Additionally, maternal behaviors in manipulated litters were compared against undisturbed litters via scoring of videotaped interactions to examine the long-term effects of pain on dam-pup interactions. Select subjects underwent neonatal brain collection (PND6) and fluorescent *in situ* hybridization (FISH) for corticotropin-releasing hormone (CRH) and the immediate early gene c-fos. Other subjects were raised to juvenile age (PND24 and PND25) and underwent innate anxiety testing utilizing an elevated plus maze (EPM) protocol. FISH indicated that neonatal pain influenced amygdalar CRH and c-fos expression, predominately in males. No significant increase in c-fos or CRH expression was observed in the hypothalamus. Additionally, neonatal pain altered anxiety behaviors independent of sex, with neonatal pain subjects showing the highest frequency of exploratory behavior. Neonatal manipulations did not alter maternal behaviors. Overall, neonatal pain drives CRH expression and produces behavioral changes in anxiety that persist until the juvenile stage.

## Significance Statement

This report expands on current rodent model research performed to assess the long-term effects of highly used neonatal intensive care unit (NICU) procedures. The NICU plays an integral role in pediatric medicine by significantly reducing infant mortality and providing necessary procedures to preterm or unwell newborns. However, procedures in the NICU are often stressful and painful. A common procedure performed in the NICU is heel lances to monitor blood composition. This, along with numerous other painful procedures, are often performed on NICU infants without the benefit of analgesics. Our study identifies key neurologic indicators which are altered in response to neonatal pain. Additionally, we explore the later anxiety of subjects exposed to neonatal pain.

## Introduction

Premature infants in the neonatal intensive care unit (NICU) may be subjected to numerous painful procedures without analgesics (Anand et al., 1999; Bhutta et al., 2002; Simons et al., 2003; Grunau et al., 2006; Anand, 2007; Carbajal et al., 2008). Exposure to these procedures is correlated with an increased risk of anxiety, mood disorders, and pain sensitivity, later in life (Grunau et al., 2006; Hermann et al., 2006). However, because infants who are in the NICU for longer are likely more ill, it is often unclear whether later behavioral changes are the result of NICU trauma or their original ailment. Rodent models can be used to distinguish between these possibilities via the use of healthy neonates that are subject to manipulations which mimic NICU treatment (Fitzgerald, 1987; Anand et al., 1999; Bhutta et al., 2002; Grunau et al., 2006; Davis et al., 2018).

Neonatal pain has long-term effects on rodents. Rodents have the capacity to perceive noxious stimuli at birth (Fitzgerald, 1987). There is a body of behavioral data which indicates that neonatal rodents exposed to repeated tactile pain exhibit reduced pain thresholds (Anand et al., 1999; Davis et al., 2018; de Carvalho et al., 2019), elevated anxiety in exploratory and social tasks (Anand et al., 1999; Anand and Scalzo, 2000; Schellinck et al., 2003), and a higher preference for alcohol as adults (Anand et al., 1999), although others find no effect on anxiety behavior (Ranger et al., 2018) or a reduction of conditioned freezing (Davis et al., 2018). Importantly, differences in outcome may be due, in part, to the nature of the pain, as studies using neonatal inflammatory pain have found reduced pain sensitivity and lower anxiety in adulthood (Bhutta and Anand, 2001; Anseloni et al., 2005), although other methodological differences likely play a role. Regardless, the neurobiological mechanisms underlying these effects remain largely unknown.

One structure likely to be affected by neonatal pain is the amygdala, which appears to be involved in many forms of negative emotionality, including innate anxiety and pain (Davis, 1992; Janak and Tye, 2015). For example, activation of the amygdala, as measured by c-fos expression (Kovács, 2008), is observed after exposure to the elevated plus maze (EPM; Silveira et al., 1993). Physical pain produces a similar activation (Merali et al., 2004). Moreover, the amygdala receives input regarding aversive stimuli and projects to regions controlling descending pain inhibition pathways, anxious and defensive behaviors, and autonomic activation. The current studies examined the effects of neonatal pain on amygdalar c-fos expression and EPM behavior.

There is also evidence that corticotropin-releasing hormone (CRH) may be involved in the long-term consequences of neonatal pain. CRH is produced in several densely populated regions of the CNS, including the central nucleus of the amygdala (CeA), paraventricular nucleus of the hypothalamus (PVN), and bed nucleus of the stria terminalis (BNST; Holsboer, 1999; Plotsky et al., 2005). Amygdalar CRH has increasingly been implicated in many forms of stress (Cratty et al., 1995; Korte and De Boer, 2003). Additionally, other studies have indicated that chronic pain (Ulrich-Lai et al., 2006) and neonatal stress (Brunson et al., 2001) elevate amygdalar, but not hypothalamic, CRH expression. Thus, the current experiments examine amygdalar CRH expression following neonatal pain.

The purpose of this study was to further our understanding of neuronal mechanisms by which neonatal pain affects later emotional responding. Additionally, our study assessed whether there were lasting effects of neonatal pain and handling on maternal behaviors. We hypothesized that our neonatal pain model would cause an increased expression of CRH mRNA in the amygdala of pups independent of maternal rearing behaviors. Furthermore, we hypothesized that downstream effects of neonatal pain would alter juvenile innate anxiety responses. To accomplish this, the current studies used c-fos and CRH fluorescent *in situ* hybridization (FISH) as a measure of nociceptive neuronal activation and CRH expression and the EPM to measure juvenile innate anxiety.

## Materials and Methods

### Subjects

Most of the subjects were bred in house from Charles River Sprague Dawley stock. These subjects were used for maternal behavior recordings, neonatal FISH, and anxiety assays. A subset of subjects was ordered as timed-pregnant dams (Charles River; used for anxiety assays) before the development of our breeding colony. Timed-pregnant females arrived on gestational day (GD)12, were singly housed, and gave birth in house on either GD21 or GD22. Our in-house breeding procedure was similar to other published works (Davis et al., 2018; Ogilvie and Rivier, 1997) and also all gave birth, singly housed on either GD21 or GD22. GD22 was considered the day of birth and called postnatal day (PND)0. Statistical analysis found no significant differences between timed-pregnant and in-house bred subjects and they were therefore combined for all further analyses.

Starting at birth, all rats were housed in 43 × 44 × 20 cm closed-environment cages (Innovive). Rats were maintained on 12/12 h light/dark cycle with lights on at 7 A.M. and were provided food and water *ad libitum*.

On PND1, litters were randomly assigned to either neonatal manipulation or an undisturbed control condition, with every litter cohort containing at least one litter of each assignment. Litters were culled to 10 rats (five males and five females when possible). Litters were weaned on PND21 and housed with same-sex littermates containing approximately five rats per cage. On completion of anxiety assays, rats were euthanized by CO_2_ asphyxiation. All rats used for FISH were euthanized immediately before neurologic tissue collection via SomnaSol pentobarbital (Henry Schein Animal Health) overdose and cardiac perfusion. All animal euthanasia was consistent with American veterinary association procedures.

A total of 16 litters were recorded for maternal behavior analysis (8 neonatal manipulation and 8 undisturbed). For RNAscope FISH experimentation, 35 rats from seven neonatally-manipulated litters and six undisturbed litters were used. Lastly, 107 rats from 13 neonatally-manipulated litters and 11 undisturbed litters were used in our anxiety assays. In all cases, no more than one same-sex littermate was assigned to any experimental condition. All animal procedures were performed in accordance with University of New England Institutional Animal Care and Use Committee and in compliance with NIH animal care and use guidelines.

### Neonatal manipulations

**Figure 1.**
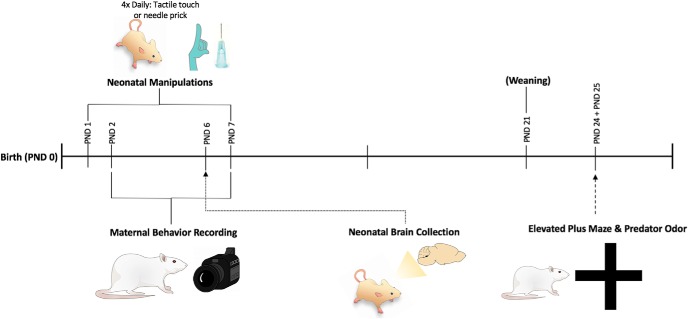
Timeline representation of animal experimentation process from birth to elevated plus maze behavioral testing. Neonatal manipulations were repeated four times daily, 2 h apart. Manipulations entailed either a tactile touch or needle prick of the left hindpaw PND1–PND7. Maternal behavior recordings occurred before the start of manipulations PND2–PND7. A subset of subjects from litters were euthanized and underwent brain collection on PND6 for FISH. Weaning of litters into same sex groupings occurred on PND21. Elevated plus maze and predator odor exposure occurred on PND24 and PND25 in a counterbalanced order by day.

Similar to manipulation procedures performed by (Davis et al., 2018; Anand et al., 1999), pups in litters designated for neonatal manipulation were marked and randomly assigned to receive either a needle prick from a 23-gauge needle tip in their left hindpaw (pain group), or a noninvasive tactile touch on the left-hind paw by the experimenter (handled group). All neonatal manipulation litters had both groups equally represented. Manipulations occurred four times per day, 2 h apart from PND1 to PND7, the exception being subjects used for FISH, which were euthanized for neurologic tissue collection on PND6, 15 min after the first manipulation period on that day see Figure 1 for experimental timeline.

### Maternal behavior

Litters randomly designated for maternal behavior observation were recorded using two GoPro HD Hero Session cameras for 1 h before the first manipulation on PND2–PND7, to examine the long-term effects of neonatal pain on dam-pup interactions in the absence of the acute effects of handling. Home cages were removed from the rack and placed on a cart with one camera mounted beside the cage and another mounted above (two camera angles per litter). Recordings for both neonatal manipulation and undisturbed litters were done simultaneously on each day of recording. Video recordings were later observed and scored by trained experimenters using Behavioral Observation Research Interactive Software (BORIS; Friard and Gamba, 2016). Scored behaviors were similar to other rodent maternal behavior studies (Caldji et al., 1998; Walker et al., 2003; LaPrairie and Murphy, 2007). These behaviors were pup licking/grooming, nursing (both passive and arched-back nursing), nest building (including burrowing), maternal self-grooming, and maternal contact (any dam to pup physical contact including licking/grooming and/or nursing). Our behavior scoring methods were adapted from Rincel et al. (2016), with behaviors scored for 10 s at the start of every 2-min interval of recorded observation, thus totaling 30 observation periods for every 1-h video. Lastly, ∼10% of videos were scored by two experimenters with resulting interrater reliability correlations of 0.803^a^ for pup licking/grooming, 0.921^b^ for nursing, 0.941^c^ nest building, 0.969^d^ maternal self-grooming, and 0.936^e^ maternal contact.

### Fluorescence *in situ* hybridization

FISH was conducted using a commercially available system [RNAscope; Advanced Cell Diagnostics (ACD)]. This technique assesses mRNA expression in a quantifiable manner. First, neonatal brain tissue was collected following intra-cardiac perfusion and post-fixed in Bouin’s fixative solution (RICCA Chem.) for 2 h before cryoprotection and refrigeration in a 30% sucrose solution. Subsequently, brains were embedded in Tissue-Tek O.C.T compound (Sakura Finetek) and flash frozen using liquid nitrogen. Brains were cryosectioned into 15-μm sections and mounted onto Superfrost plus microscope slides (VWR). Tissue sections were stored for approximately one month at –80°C before undergoing the FISH procedure.

The FISH used probes targeting CRH (product number: 318931) and c-fos (product number: 403591-C2). The protocol was developed using RNAscope Multiplex Fluorescent Reagent Kit v2 user manual (document number: 323100-USM), manufacturer technical note regarding tissue detachment, and manufacturer modifications for fixed frozen tissue (ACD). In the final steps of the protocol, DAPI was applied to brain sections as a counterstain for amygdala and hypothalamic regional identification.

**Figure 2.**
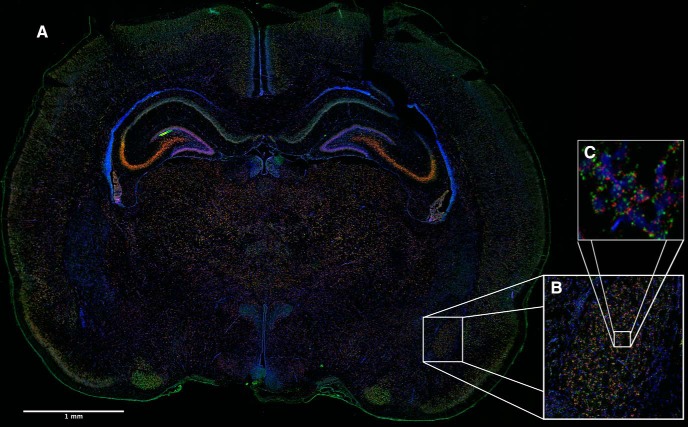
Optimized positive control section of RNAscope FISH in a PND6 rat brain. Green (channel 1): staining Polr2a, red (channel 2): staining PPIB, blue (DAPI). (***A***) complete coronal section, (***B***) zoomed in section of the amygdala, (***C***) zoomed in region of the amygdala to demonstrate punctate staining pattern.

RNAscope assays were conducted in batches consisting of tissue collected on the same date from the same litters to limit variance in animal life experience, tissue treatment, and target degradation variability. Tissue from all three conditions (pain, handled, and undisturbed) and both sexes were included in each batch. Each batch also contained additional tissue processed with either a positive control probe [product number: 320891; containing Polr2a (channel 1), PPIB (channel 2)] or negative control probe [product number: 320871; containing DapB gene accession EF191515 from the SMY strain of *Bacillus subtilis* (channels 1 and 2)]. Per manufacturer instructions (RNAscope; ACD), these controls were to ensure consistency and procedural accuracy between subsequent FISH batches. See (Fig. 2) for optimized positive control image example. All negative probe images demonstrated no specific staining.

**Figure 3.**
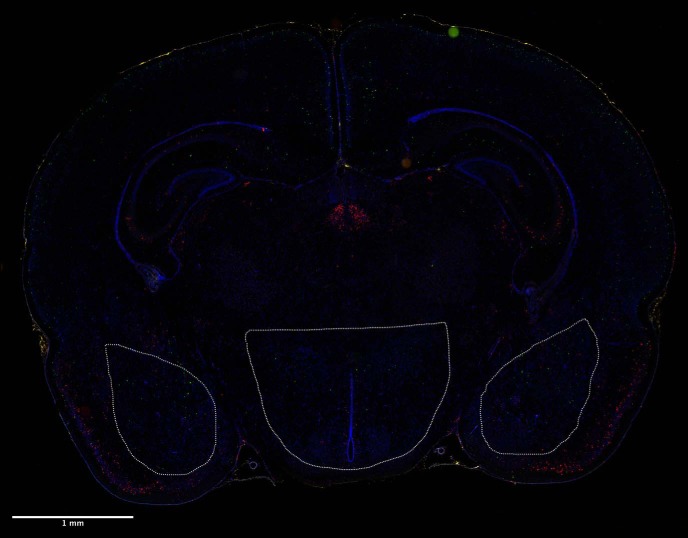
Representative coronal brain section depicting analyzed regions of interest (amygdalae and hypothalamus). Subject depicted is a male from the pain condition. CRH (green), c-fos (red), DAPI (blue).

Fluorescence multiplex imaging was done between 2 d and two weeks after FISH. All images for quantification were taken under the same magnification (20×) and exposure settings (1/25 s) for quantified channels (CRH and c-fos) on a Keyence BZ-X710 microscope. The DAPI channel exposures varied somewhat between sections due to the inherent variability in DAPI application. Sections were overlaid and stitched for analysis using image merge software provided with Keyence BZ-X710 microscope (Keyence). See Figure 3 for a sample picture with identified regions of interest.

Images were analyzed using the FIJI package of NIH’s open source image analysis software ImageJ (Schindelin et al., 2012; Schneider et al., 2012). Overlaid images were first subject to color separation and the DAPI channel was used to identify amygdala and hypothalamic regions of interest. CRH and c-fos expression were measured in our regions of interest for percentage area expressing the signal (percentage area), expression intensity (measured by Otsu threshold grayscale values), and instances of expression colocalization.

Values from the right and left regions of interest in individual brain sections were combined to generate a value of the average percentage area expressing the signal (percentage area), average expression intensity (intensity), and summation of colocalization occurrences (colocalization) for each subject. Further, a “baseline value” was calculated by taking the average of percentage area for undisturbed animals of each sex. This baseline value was then used to calculate a percentage difference score from undisturbed in both sexes via the equation: (area expressing target probe in the subject – baseline value)/baseline value × 100. The resulting values were referred to as percentage difference in area. This was done for both CRH and c-fos expression in both the amygdala and hypothalamus.

### Anxiety assays

After weaning (on PND24 and PND25), subjects were removed from their home cage, weighed, and placed in individual transport cages. Subjects were transported to the testing room and underwent a 10-min habituation period before behavioral testing to assess innate anxiety levels using an EPM and predator odor exposure in a counterbalanced by day manner.

### Elevated Plus Maze

Our EPM apparatus comprised two open arms (51 × 10 cm) and two closed arms (51 × 10 cm) connected via a center platform (10 × 10 cm). The apparatus was 49.5 cm above floor level, and constructed from wood. The only lighting in the room was a dimly lit, incandescent floor standing lamp. Before each subject was tested, the EPM was cleaned with 70% EtOH. Subjects were placed in the center of the apparatus facing an open arm and were allowed to explore freely for 5 min. The experimenter was outside of the testing room for the duration of the test. Activity was recorded and tracked using EthoVision XT (Noldus) software. Four measures were assessed: open-arm entries, open-arm time, total distance traveled, and total arm entries.

### Predator odor

Filter paper containing either two drops of 1% peppermint oil or ∼0.1-ml red fox urine (trap shack company; via Amazon.com) was placed inside an open Petri dish, and subsequently placed on opposite sides of a vent toped (44 × 23.5 cm) shoebox cage. The shoebox cage was placed inside a fume hood with the fan and lights on during the duration of the test. Rats were placed in the center of the cage facing away from the experimenter, and a real-time log of the subject’s activity was recorded for 10 min. The cage was cleaned with sparkleen 1 (Fisher Scientific) between each test. Fox urine was naturally derived and stored at room temperature in an air tight container (in lab) when not in use.

Despite pilot data to the contrary, all rats demonstrated a common strong side preference (likely due to some unknown characteristic of the fume hood) rather than a specific reaction to the odors. Therefore, the predator odor experimental data were not analyzed further, although the EPM data from these rats was retained.

### Statistical analysis

All data (maternal behavior, FISH, and anxiety assays) were analyzed with IBM SPSS version 22 mixed model ANOVAs and Tukey’s *post hoc* analysis were performed where applicable (See Table 1). Maternal behavior statistical analysis consisted of 2 (litter type: neonatal manipulation, undisturbed) × 6 (PND2–PND6) repeated measures MANOVAs with testing day serving as the repeated variable.

**Table 1.** Statistical table defining type of test and observed power for each statistical analysis

Test	Data structure	Type of test	Power or confidence interval (lower/upper)
a	Presumed normal	Within item interclass correlation; interrater reliability test	Lower: 0.67/upper: 0.88
b	Presumed normal	Within item interclass correlation; interrater reliability test	Lower: 0.87/upper: 0.95
c	Presumed normal	Within item interclass correlation; interrater reliability test	Lower: 0.90/upper: 0.96
d	Presumed normal	Within item interclass correlation; interrater reliability test	Lower: 0.95/upper: 0.98
e	Presumed normal	Within item interclass correlation; interrater reliability test	Lower: 0.89/upper: 0.96
f	Presumed normal	2 × 6 repeated measures MANOVA	Power: 0.067
g	Presumed normal	2 × 6 repeated measures MANOVA	Power: 0.085
h	Presumed normal	2 × 6 repeated measures MANOVA	Power: 0.276
i	Presumed normal	2 × 6 repeated measures MANOVA	Power: 0.112
j	Presumed normal	2 × 6 repeated measures MANOVA	Power: 0.073
k	Presumed normal	2 × 6 repeated measures MANOVA	Power: 0.680
l	Presumed normal	2 × 6 repeated measures MANOVA; *t* test for condition by day	Lower: 1.82/upper: 9.18
m	Presumed normal	2 × 6 repeated measures MANOVA; *t* test for condition by day	Lower: –4.84/upper: 0.34
n	Presumed normal	2 × 6 repeated measures MANOVA	Power: 0.956
o	Presumed normal	2 × 6 repeated measures MANOVA	Power: 1.000
p	Presumed normal	2 × 6 repeated measures MANOVA	Power: 0.857
q	Presumed normal	2 × 2 × 3 ANOVA	Power: 0.761
r	Presumed normal	2 × 2 × 3 ANOVA	Power: 0.451
s	Presumed normal	2 × 2 × 3 ANOVA	Power: 0.080
t	Presumed normal	2 × 2 × 3 ANOVA	Power: 0.210
u	Presumed normal	2 × 2 × 3 ANOVA	Power: 0.353
v	Presumed normal	2 × 2 × 3 ANOVA	Power: 0.058
w	Presumed normal	2 × 2 × 3 ANOVA	Power: 0.088
x	Presumed normal	2 × 2 × 3 ANOVA	Power: 0.119
y	Presumed normal	ANOVA	Power: 0.704
z	Presumed normal	2 × 3 ANOVA	Power: 0.385
aa	Presumed normal	2 × 3 ANOVA	Power: 0.474
ab	Presumed normal	2 × 3 ANOVA	Power: 0.486
ac	Presumed normal	2 × 3 ANOVA	Power: 0.714
ad	Presumed normal	2 × 3 ANOVA	Power: 0.455
ae	Presumed normal	2 × 3 ANOVA	Power: 0.314
af	Presumed normal	2 × 3 ANOVA	Power: 0.431
ag	Presumed normal	2 × 3 ANOVA	Power: 0.053
ah	Presumed normal	2 × 3 ANOVA	Power: 0.185
ai	Presumed normal	2 × 3 ANOVA	Power: 0.064
aj	Presumed normal	2 × 3 ANOVA	Power: 0.077
ak	Presumed normal	2 × 3 ANOVA	Power: 0.057
al	Presumed normal	2 × 3 ANOVA	Power: 0.660
am	Presumed normal	2 × 3 ANOVA; Tukey’s *post hoc*	Lower: 0.386/upper: 40.01
an	Presumed normal	2 × 3 ANOVA; Tukey’s *post hoc*	Lower: –2.35/upper: 35.51
ao	Presumed normal	2 × 3 ANOVA	Power: 0.423
ap	Presumed normal	2 × 3 ANOVA	Power: 0.390
aq	Presumed normal	2 × 3 ANOVA	Power: 0.071
ar	Presumed normal	2 × 3 ANOVA	Power: 0.053
as	Presumed normal	2 × 3 ANOVA	Power: 0.110
at	Presumed normal	ANOVA; Tukey’s *post hoc*	Lower: 8.52/upper: 123.84
au	Presumed normal	ANOVA; Tukey’s *post hoc*	Lower: –10.84/upper: 92.30
av	Presumed normal	ANOVA	Power: 0.139
aw	Presumed normal	ANOVA	Power: 0.348
ax	Presumed normal	ANOVA	Power: 0.157
ay	Presumed normal	ANOVA	Power: 0.999
az	Presumed normal	ANOVA; Tukey’s *post hoc*	Lower: 16.90/upper: 46.76
ba	Presumed normal	ANOVA; Tukey’s *post hoc*	Lower: 14.64/upper: 48.03
bb	Presumed normal	ANOVA	Power: 0.106
bc	Presumed normal	ANOVA	Power: 0.133
bd	Presumed normal	ANOVA	Power: 0.094
be	Presumed normal	ANOVA	Power: 0.061
bf	Presumed normal	ANOVA	Power: 0.071
bg	Presumed normal	ANOVA	Power: 0.064
bh	Presumed normal	ANOVA	Power: 0.143
bi	Presumed normal	2 × 2 × 3 ANOVA	Power: 0.580
bj	Presumed normal	2 × 2 × 3 ANOVA	Power: 0.098
bk	Presumed normal	2 × 2 × 3 ANOVA	Power: 0.212
bl	Presumed normal	2 × 2 × 3 ANOVA	Power: 0.056
bm	Presumed normal	2 × 2 × 3 ANOVA	Power: 0.145
bn	Presumed normal	2 × 2 × 3 ANOVA	Power: 0.071
bo	Presumed normal	2 × 2 × 3 ANOVA	Power: 0.136
bp	Presumed normal	2 × 2 × 3 ANOVA	Power: 0.264
bq	Presumed normal	2 × 2 × 3 ANOVA	Power: 0.231
br	Presumed normal	2 × 2 × 3 ANOVA	Power: 0.178
bs	Presumed normal	2 × 2 × 3 ANOVA	Power: 0.172
bt	Presumed normal	2 × 2 × 3 ANOVA	Power: 0.144
bu	Presumed normal	2 × 2 × 3 ANOVA	Power: 0.173
bv	Presumed normal	2 × 2 × 3 ANOVA	Power: 0.256
bw	Presumed normal	2 × 2 × 3 ANOVA	Power: 0.588
bx	Presumed normal	2 × 2 × 3 ANOVA	Power: 0.269
by	Presumed normal	2 × 2 × 3 ANOVA	Power: 0.189
bz	Presumed normal	2 × 2 × 3 ANOVA	Power: 0.065
ca	Presumed normal	2 × 2 × 3 ANOVA	Power: 0.198
cb	Presumed normal	2 × 2 × 3 ANOVA	Power: 0.122
cc	Presumed normal	2 × 2 × 3 ANOVA	Power: 0.244
cd	Presumed normal	2 × 2 × 3 ANOVA	Power: 0.431
ce	Presumed normal	2 × 2 × 3 ANOVA	Power: 0.086
cf	Presumed normal	2 × 2 × 3 ANOVA	Power: 0.358
cg	Presumed normal	2 × 2 × 3 ANOVA	Power: 0.063
ch	Presumed normal	2 × 2 × 3 ANOVA	Power: 0.209
ci	Presumed normal	2 × 2 × 3 ANOVA	Power: 0.085
cj	Presumed normal	2 × 2 × 3 ANOVA	Power: 0.329
ck	Presumed normal	ANOVA	Power: 0.640
cl	Presumed normal	ANOVA; Tukey’s *post hoc*	Lower: 0.72/upper: 23.33
cm	Presumed normal	ANOVA; Tukey’s *post hoc*	Lower: –2.55/upper: 21.71
cn	Presumed normal	ANOVA; Tukey’s *post hoc*	Lower: –9.54/upper: 14.43
co	Presumed normal	ANOVA	Power: 0.557
cp	Presumed normal	ANOVA	Power: 0.180
cq	Presumed normal	ANOVA	Power: 0.377

Statistical data pertaining to each test is indicated by superscript letters within text.

RNAscope FISH statistical analysis consisted of first a 2 (amygdala side: right, left) × 2 (sex: male, female) × 3 (neonatal condition: undisturbed, handled, and pain) ANOVA to assess for staining lateralization in the CRH channel. When no staining lateralization was found, 2 (sex: male, female) × 3 (neonatal condition: undisturbed, handled, and pain) ANOVAs were conducted for each measure regarding CRH and c-fos (percentage difference in area, sum of colocalization, and Otsu threshold grayscale value). As sex differences were common, follow-up one-way ANOVAs and Tukey’s HSD *post hoc* tests were used. Guidance from ACD suggests that number of positive straining events (operationalized here as area), rather than brightness, is the best method to assess differences in RNAscope expression.

Statistical analysis for anxiety assays consisted of a 2 (testing order: predator exposure on PND24, PND25) × 2 (sex: male, female) × 2 (breeding condition) × 3 (neonatal condition: undisturbed, handled, pain) ANOVA comparing average time in open arms, number of open arm entries, number of total arm entries, and total distance traveled across each group. Follow-up ANOVAs and Tukey’s *post hoc* tests were used as appropriate.

## Results

### Experiment 1: maternal behavior

Dams of litters that experienced neonatal manipulations and dams of undisturbed litters did not differ in their behaviors toward pups as shown by 2 (litter type: neonatal manipulation, undisturbed) × 6 (day: PND2–PND7) repeated measures MANOVAs with testing day serving as the repeated variable. Condition alone had no effect on any maternal behavior measure including the following: pup licking/grooming *F*_(1,14)_ = 0.170, *p* = 0.686, partial η^2^ = 0.012^f^; nursing *F*_(1,14)_ = 0.343, *p* = 0.568, partial η^2^ = 0.024 × *g*; nest building *F*_(1,14)_ = 2.14, *p* = 0.166, partial η^2^ = 0.133^h^; maternal self-grooming *F*_(1,14)_ = 0.604, *p* = 0.450, partial η^2^ = 0.041^i^; and maternal contact *F*_(1,14)_ = 0.230, *p* = 0.639, partial η^2^ = 0.016^j^.

**Figure 4.**
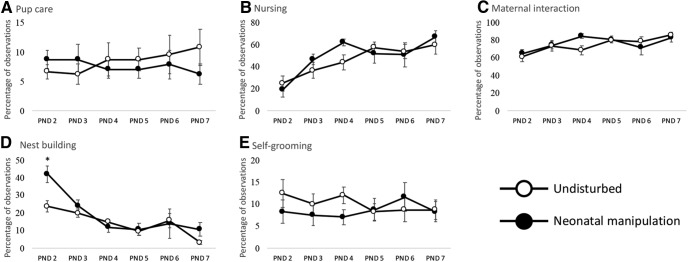
Maternal behaviors recorded from PND2 to PND7 for undisturbed and manipulation litters. Each data point represents the mean of eight dams/litters for percentage of observed maternal behaviors. Recorded behaviors were (***A***) pup care, (***B***) nursing, (***C***) maternal interaction, (***D***) nest building, and (***E***) self-grooming. Error bars represent ±SEM; * denotes significant (*p* < 0.05) difference between neonatal manipulation and undisturbed litters for that day and behavior.

There was a significant condition × day interaction for nest building *F*_(5,10)_ = 3.37, *p* = 0.048, partial η^2^ = 0.628^k^ suggesting that time spent nest building differed in the neonatal manipulation litters compared to undisturbed litters (Fig. 4). Subsequent *t* tests examining the effect of condition on each day found a significant difference on PND2 *p* = 0.006; CI = 1.821 – 9.179^l^ and a trend toward a significant difference on PND7 *p* = 0.083; CI = –4.837 – 0.337^m^; with neonatal manipulation litters having more nest building on both days (Fig. 4). In addition, there was a main effect of day for nursing *F*_(5,10)_ = 7.06, *p* = 0.005, partial η^2^ = 0.779^n^; nest building *F*_(5,10)_ = 24.46, *p* < 0.001, partial η^2^ = 0.924^o^; and maternal interaction *F*_(5,10)_ = 4.98, *p* = 0.015, partial η^2^ = 0.714^p^. These data suggest that maternal behaviors change over development but are not affected by our acute repeated neonatal manipulations.

### Experiment 2: Fluorescence *in situ* hybridization

Two subjects (male undisturbed and female handled) were excluded as statistical outliers for having CRH expression data >2.5 SDs from their respective group means in the amygdala. For the hypothalamus experiments, two subjects (male handled and female handled) were excluded for having CRH expression data >2.5 SDs from their respective group means.

**Figure 5.**
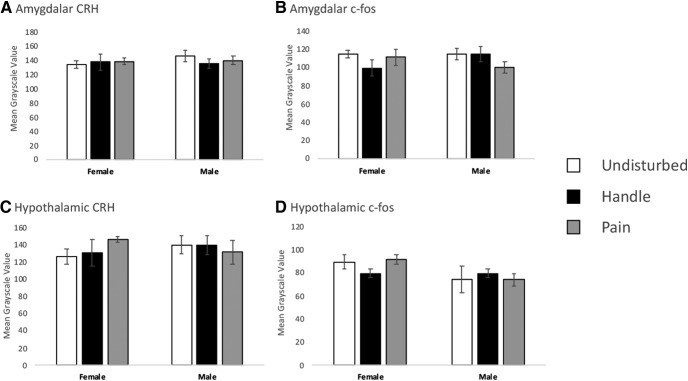
Amygdalar and hypothalamic FISH luminance measures for CRH and c-fos in PND6 rats. Luminance was measured for all regions via mean grayscale value after Otsu threshold application. All regions and corresponding subjects used in our CRH and c-fos percentage difference in area measurements were also used in these luminance measurements. Average greyscale values are shown for: ***A*** CRH expression in the amygdala, ***B*** c-fos expression in the amygdala, ***C*** CRH expression in the hypothalalmus, ***D*** c-fos expression in the hypothalamus. Data are presented as means with error bars as ±SEM.

There were no observed differences (sex, condition, or interaction) in Otsu threshold mean grayscale values for all measures of CRH and c-fos in amygdalar and hypothalamic regions (Fig. 5). These finding are consistent with standard RNAscope quantification procedures.

Unexpectedly, sex differences were apparent both in the pattern of results and in most statistical analyses. Therefore, we separated all subsequent analyses for this experiment by sex.

### Lateralization of the amygdala

An initial 2 (amygdala side: right, left) × 2 (sex: male, female) × 3 (neonatal condition: undisturbed, handled, and pain) ANOVA resulted in a significant main effect of hemisphere *F*_(1,27)_ = 7.66, *p* = 0.010, partial η^2^ = 0.221^q^ for percentage area expressing CRH. The left amygdala (mean = 0.101, SEM = 0.006) exhibiting a greater area expressing CRH compared to right (mean = 0.085, SEM = 0.005). Additionally, we observed a trend toward an interaction between hemisphere × sex *F*_(1,27)_ = 3.63, *p* = 0.068, partial η^2^ = 0.118^r^ for percentage area expressing CRH. Male left amygdala (mean = 0.105, SEM = 0.010) showing the highest percentage area expressing CRH followed by female left amygdala (mean = 0.096, SEM = 0.009), female right amygdala (mean = 0.091, SEM = 0.008), and male right amygdala (mean = 0.079, SEM = 0.007). There were no significant hemisphere × condition interaction *F*_(2,27)_ = 0.211, *p* = 0.811, partial η^2^ = 0.015^s^ nor a hemisphere × sex × condition interaction *F*_(2,27)_ = 1.026, *p* = 0.372, partial η^2^ = 0.071^t^ for percentage area expressing CRH.

Additionally, a 2 (amygdala side: right, left) × 2 (sex: male, female) × 3 (neonatal condition: undisturbed, handled, and pain) ANOVA found no main effects or interactions in the lateralization of c-fos expression for: hemisphere *F*_(1,27)_ = 2.687, *p* = 0.113, partial η^2^ = 0.090^u^; hemisphere × sex *F*_(1,27)_ = 0.073, *p* = 0.788, partial η^2^ = 0.003^v^; hemisphere × condition *F*_(2,27)_ = 0.267, *p* = 0.768, partial η^2^ = 0.019^w^; or hemisphere × sex × condition *F*_(2,27)_ = 0.473, *p* = 0.628, partial η^2^ = 0.034^x^.

As this demonstrated no lateralized effect of condition for CRH or c-fos expression, all further amygdala analyses were collapsed across hemispheres.

### Effects of sex: CRH and c-fos expression and colocalization

There was a large difference in pattern of amygdala activation depending on the sex of the subject. In regards to amygdalar CRH expression, we observed a trend toward a significant main effect of sex *F*_(1,27)_ = 2.986, *p* = 0.095, partial η^2^ = 0.100^y^, condition *F*_(2,27)_ = 2.601, *p* = 0.093, partial η^2^ = 0.162^z^, and the interaction of sex × condition *F*_(2,27)_ = 2.677, *p* = 0.087, partial η^2^ = 0.165^aa^ on percentage difference in area expressing CRH in the amygdala. Indicating that males and females exhibited different patterns of amygdalar CRH expression in response to neonatal pain.

In our amygdalar c-fos measures, there was a significant main effect of sex *F*_(1,27)_ = 6.86, *p* = 0.014, partial η^2^ = 0.202^ab^ in the percentage difference in area of the amygdala expressing c-fos: females (mean = 0.54%, SEM = 10.98%) males (mean = 84.22%, SEM = 27.80%). However, this was not the case for the effect of condition *F*_(2,27)_ = 2.48, *p* = 0.103, partial η^2^ = 0.155^ac^, nor the interaction of sex × condition *F*_(2,27)_ = 1.63, *p* = 0.215, partial η^2^ = 0.108^ad^ for percentage area of the amygdala expressing c-fos. Indicating that males and females exhibited different patterns of neuronal activity in response to neonatal pain.

Furthermore, there was a trend toward a significant main effect of sex *F*_(1,26)_ = 3.44, *p* = 0.075, partial η^2^ = 0.117^ae^ in the percentage difference in area expressing CRH in the hypothalamus. Suggesting that males and females exhibited different patterns of hypothalamic CRH expression in response to neonatal pain. This was not the case for the main effect of condition *F*_(2,26)_ = 0.022, *p* = 0.978, partial η^2^ = 0.002^af^ nor the sex × condition interaction *F*_(2,26)_ = 0.883, *p* = 0.426, partial η^2^ = 0.064^ag^ for percentage difference in area expressing CRH in the hypothalamus. Additionally, we observed no effects or trends in the percentage difference in area expressing c-fos for the hypothalamus; sex *F*_(1,26)_ = 0.132, *p* = 0.720, partial η^2^ = 0.005^ah^; condition *F*_(2,26)_ = 0.194, *p* = 0.825, partial η^2^ = 0.015^ai^; and sex × condition *F*_(2,26)_ = 0.052, p = 0.949, partial η^2^ = 0.004^aj^.

There was a difference in the pattern of amygdala and hypothalamus c-fos and CRH mRNA expression depending on the sex of the subject. Preliminary CRH and c-fos colocalization analysis further exemplifies this sex difference. For amygdalar CRH and c-fos colocalization, there was a significant main effect of condition *F*_(2,27)_ = 3.958, *p* = 0.031, partial η^2^ = 0.227^ak^. Subsequent Tukey’s *post hoc* tests found that pain subjects (mean = 53, SEM = 5.62) had significantly more colocalization then undisturbed subjects (mean = 32.8, SEM = 4.27) *p* = 0.045; CI = 0.386 – 40.014^al^ and only trended toward significantly more than handled subjects (mean = 36.42, SEM = 7.02) *p* = 0.094; CI = –2.346 – 35.513^am^. Additionally, there was a trend in the main effect of sex for amygdalar CRH and c-fos colocalization *F*_(1,27)_ = 3.348, *p* = 0.078, partial η^2^ = 0.110^an^, but not in the interaction of sex × condition *F*_(2,27)_ = 2.080, *p* = 0.145, partial η^2^ = 0.133^ao^. Further indicating that males and females react differently to neonatal adversity while also exemplifying differences between neonatal conditions.

There was no observed effects or interactions in the preliminary analysis of hypothalamic CRH and c-fos colocalization; condition *F*_(2,26)_ = 0.155, *p* = 0.857, partial η^2^ = 0.012^ap^, sex *F*_(1,26)_ = 0.030, *p* = 0.863, partial η^2^ = 0.001^aq^, sex × condition *F*_(2,26)_ = 0.413, *p* = 0.666, partial η^2^ = 0.031^ar^.

To conclude, our analysis of CRH and c-fos expression and colocalization within the amygdala and hypothalamus, exemplifies that CRH mRNA expression and neuronal activity (measured by c-fos mRNA expression) are sex dependent.

### Effects of neonatal pain on the amygdala

**Figure 6.**
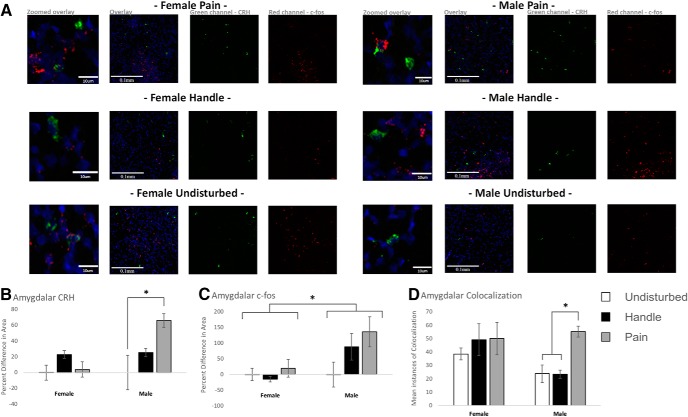
Amygdalar quantification of FISH for transcription products CRH and c-fos. Examining the amygdala following acute neonatal manipulations [pain (*N* = 5 female, *N* = 6 male), handled (*N* = 6 female, *N* = 6 male), and undisturbed (*N* = 6 female, *N* = 4 male)] in PND6 rats. ***A***, Representative amygdala segments for all neonatal manipulation groups with corresponding channel separation panels for CRH (green) and c-fos (red). DAPI was used as a counter stain for regional identification (blue). The first panel of each group is a zoomed snapshot of the corresponding overlay panel, whereas the next three panels represent a wider-field view of the overlay, CRH and c-fos, respectively. ***B***, CRH expression in handled and pain conditions as a percentage difference in area expressed from their same sex undisturbed counterpart. ***C***, c-fos expression in handled and pain conditions as a percentage difference in area expressed from their same sex undisturbed counterpart. ***D***, colocalization of CRH and c-fos expression. For ***B–D***, data are presented as means with error bars as ±SEM; * denotes significant (*p* < 0.05) difference between indicated groups.

Neonatal pain enhanced amygdalar CRH expression in male, but not female, subjects. There was a significant main effect of condition *F*_(2,13)_ = 4.93, *p* = 0.026, partial η^2^ = 0.431^as^ in percentage difference in area expressing CRH in male amygdalar tissue (Fig. 6). Subsequent *post hoc* tests indicated a significant difference between male pain and undisturbed (*p* = 0.024; CI 8.52 – 123.84)^at^ but not handled subjects (*p* = 0.132; CI = –10.84 – 92.30)^au^. There was no observed effect of condition *F*_(2,14)_ = 0.658, *p* = 0.533, partial η^2^ = 0.086^av^ in percentage difference in area expressing CRH in female amygdalar tissue.

Neonatal pain did not significantly affect amygdalar c-fos expression, despite an apparently similar trend with male CRH expression (Fig. 6). There was no observed effect of condition in percentage difference in area expressing c-fos for male *F*_(2,13)_ = 2.07, *p* = 0.166, partial η^2^ = 0.241^aw^ or female *F*_(2,14)_ = 0.784, *p* = 0.476, partial η^2^ = 0.101^ax^ amygdala regions.

Neonatal pain increased amygdalar colocalization of CRH and c-fos in male, but not female, subjects. For male subjects, there was a significant main effect of condition *F*_(2,13)_ = 19.57, *p* < 0.001, partial η^2^ = 0.751^ay^ for amygdalar CRH and c-fos colocalization (Fig. 6). Subsequent *post hoc* tests indicated significantly higher levels of male amygdalar colocalization after pain compared to both handled (*p* < 0.001; CI = 16.904 – 46.763)^az^ and undisturbed conditions (*p* = 0.001; CI = 14.642 – 48.025)^ba^. As was the case with CRH expression, females showed no significant effect of condition *F*_(2,14)_ = 0.429, *p* = 0.660, partial η^2^ = 0.058^bb^.

### Effects of neonatal pain on the hypothalamus

**Figure 7.**
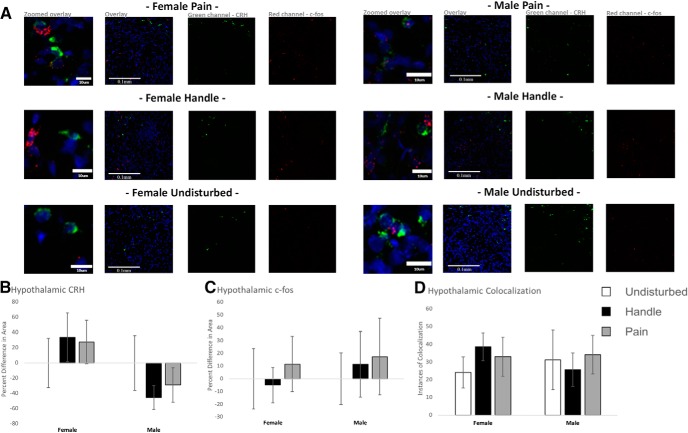
Hypothalamic quantification of FISH for transcription products CRH and c-fos. Examining hypothalamic expression following acute neonatal manipulations [pain (*N* = 5 female, *N* = 7 male), handled (*N* = 6 female, *N* = 4 male), and undisturbed (*N* = 6 female, *N* = 4 male)] in PND6 rats. ***A***, Representative hypothalamus segments for all neonatal manipulation groups with corresponding channel separation panels for CRH (green) and c-fos (red). DAPI was used as a counter stain for regional identification (blue). The first panel of each group is a zoomed snapshot of the corresponding overlay panel, whereas the next three panels represent a wider-field view of the overlay, CRH and c-fos, respectively. ***B***, CRH expression in handled and pain conditions as a percentage difference in area expressed from their same sex undisturbed counterpart. ***C***, c-fos expression in handled and pain conditions as a percentage difference in area expressed from their same sex undisturbed counterpart. ***D***, Colocalization of CRH and c-fos expression. For ***B–D***, data are presented as means with error bars as ±SEM.

Neonatal pain had minimal effects on hypothalamic expression of CRH, c-fos, and its colocalization (Fig. 7). When separated by sex, neonatal pain did not significantly affect male *F*_(1,12)_ = 0.640, *p* = 0.544, partial η^2^ = 0.096^bc^; or female hypothalamic CRH expression *F*_(2,14)_ = 0.341, *p* = 0.717, partial η^2^ = 0.046^bd^.

Neonatal pain had no statistically significant effect on hypothalamic c-fos mRNA expression nor CRH and c-fos mRNA colocalization. Male hypothalamic c-fos expression *F*_(2,12)_ = 0.090, *p* = 0.914, partial η^2^ = 0.015^be^. The same was found for female hypothalamic c-fos expression *F*_(2,14)_ = 0.166, *p* = 0.849, partial η^2^ = 0.023^bf^. For hypothalamic CRH and c-fos colocalization, male colocalization: *F*_(2,12)_ = 0.113, *p* = 0.894, partial η^2^ = 0.019^bg^; and female colocalization: *F*_(2,14)_ = 0.684, *p* = 0.521, partial η^2^ = 0.089^bh^.

### Experiment 3: EPM and predator odor anxiety assays

Outlier values were excluded from analysis that had a value that differed by >2.5 SDs from their respective group mean in any given measure (no >2 for any measure in any experimental group).

On PND24 and PND25, subjects were exposed to the EPM and predator odor testing in a counterbalanced order by day. As mentioned above (see Materials and Methods), due to methodological difficulties with the task (all subjects preferred the same side of the chamber, regardless of odor placement), predator odor data were not analyzed further. Nevertheless, it is still treated as an independent variable in our EPM experiment.

An initial 3 (condition: undisturbed, handled, pain) × 2 (sex) × 2 (order of testing; predator odor or EPM first) ANOVA was conducted on each dependent measure (reported below). As no significant differences due to sex or order of testing was found for any dependent measure, further analyses were conducted examining the effects of condition when collapsed across these variables.

### Effects of sex and testing order

For open arm time, there was a nearly significant effect of condition *F*_(2,91)_ = 3.073, *p* = 0.051 partial η^2^ = 0.063^bi^. There were no other significant effects or interactions. This includes the main effect of sex: *F*_(1,91)_ = 0.419, *p* = 0.519, partial η^2^ = 0.005^bj^; and main effect of order: *F*_(1,91)_ = 1.371, *p* = 0.245, partial η^2^ = 0.015^bk^. There were also no significant interactions including sex × order: *F*_(1,91)_ = 0.051, *p* = 0.822, partial η^2^ =0.001^bl^; sex × condition: *F*_(2,91)_ = 0.586, *p* = 0.559, partial η^2^ = 0.013^bm^; order × condition: *F*_(2,91)_ = 0.141, *p* = 0.868, partial η^2^ = 0.003^bn^; and sex × order × condition: *F*_(2,91)_ = 0.533, *p* = 0.588, partial η^2^ = 0.012^bo^.

For open arm entries, there were no significant effects or interactions. This includes the main effect of sex: *F*_(1,91)_ = 1.797, *p* = 0.183, partial η^2^ = 0.019^bp^; main effect of order: *F*_(1,91)_ = 1.527, *p* = 0.220, partial η^2^ = 0.017^bq^; and main effect of condition: *F*_(2,91)_ = 0.775, *p* = 0.464, partial η^2^ = 0.017^br^. There were also no significant interactions including sex × order: *F*_(1,91)_ = 1.041, *p* = 0.310, partial η^2^ = 0.011^bs^; sex × condition: *F*_(2,91)_ = 0.580, *p* = 0.562, partial η^2^ = 0.013^bt^; order × condition: *F*_(2,91)_ = 0.745, *p* = 0.478, partial η^2^ = 0.016^bu^; and sex × order × condition: *F*_(2,91)_ = 1.198, *p* = 0.307, partial η^2^ = 0.026^bv^.

For total distance traveled there was a main effect of condition: *F*_(2,95)_ = 3.124, *p* = 0.049, partial η^2^ = 0.062^bw^, but no other significant effects or interactions. This includes the main effect of sex: *F*_(1,95)_ = 1.839, *p* = 0.178, partial η^2^ = 0.019^bx^; and main effect of order: *F*_(1,95)_ = 1.174, *p* = 0.281, partial η^2^ = 0.012^by^. There were also no significant interactions including sex × order: *F*_(1,95)_ = 0.137, *p* = 0.713, partial η^2^ = 0.001^bz^; sex × condition: *F*_(2,95)_ = 0.883, *p* = 0.417, partial η^2^ = 0.018^ca^; order × condition: *F*_(2,95)_ = 0.452, *p* = 0.637, partial η^2^ = 0.009^cb^; and sex × order × condition: *F*_(2,95)_ = 1.134, *p* = 0.326, partial η^2^ = 0.023^cc^.

For total arm entries there was a trend toward a main effect of order: *F*_(1,92)_ = 3.259, *p* = 0.074, partial η^2^ = 0.034^cd^, reflecting a tendency for previously stressed rats to make more transitions, but no other significant effects or interactions. This includes the main effect of sex: *F*_(1,92)_ = 0.314, *p* = 0.576, partial η^2^ = 0.003^ce^; and main effect of condition: *F*_(2,92)_ = 1.748, *p* = 0.180, partial η^2^ = 0.037^cf^. There were also no significant interactions including sex × order: *F*_(1,92)_ = 0.114, *p* = 0.736, partial η^2^ =0.001^cg^; sex × condition: *F*_(2,92)_ = 0.943, *p* = 0.363, partial η^2^ = 0.020^ch^; order × condition: *F*_(2,92)_ = 0.229, *p* = 0.795, partial η^2^ = 0.005^ci^; and sex × order × condition: *F*_(2,92)_ = 1.588, *p* = 0.210, partial η^2^ = 0.033^cj^.

As no significant (or trending) effects of sex, testing order or breeding were found, all subsequent analyses were conducted as one-way ANOVAs examining the effects of condition.

### Effects of condition on elevated plus maze behavior

**Figure 8.**
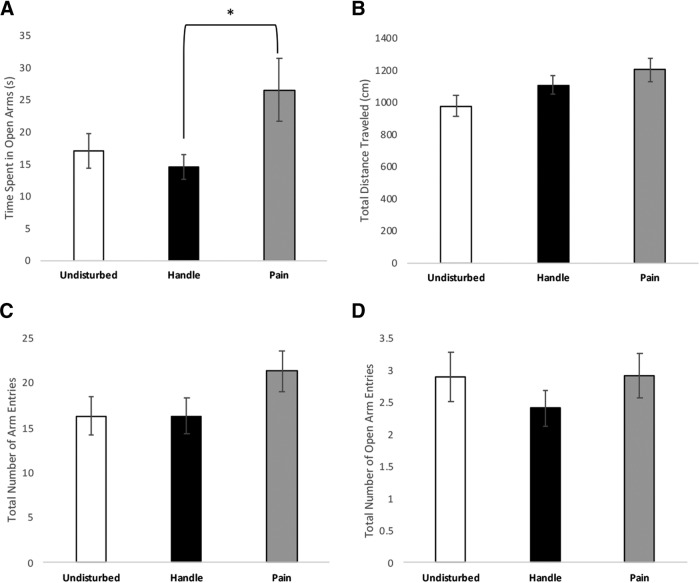
Effects of neonatal pain or handling on explorative and anxiety-like behavior in PND24 and PND25 rats using elevated plus maze anxiety test. *N*s ranged from 29 to 38 per condition among all analyzed behaviors. The elevated plus maze data were measured and compared by (***A***) time spent in open arms, (***B***) total distance traveled, (***C***) total number of arm entries, and (***D***) total number of open arm entries. Data are presented as means with error bars as ±SEM; * denotes significant (*p* < 0.05) difference between indicated groups.

When collapsed across the irrelevant variables, one-way ANOVAs reveal that neonatal pain affected time spent exploring open arms, as demonstrated by a significant effect of condition *F*_(2,100)_ = 3.49, *p* = 0.034, partial η^2^ = 0.065^ck^ (Fig. 8). Tukey’s *post hoc* tests demonstrate a significant difference between the neonatal pain and handled groups (*p* = 0.03; CI = 23.33 – 0.72)^cl^, but not between the pain and undisturbed groups (*p* = 0.15; CI = –2.55 – 21.71)^cm^ or the handled and undisturbed groups (*p* = 0.88; CI = –9.54 – 14.43^cn^; Fig. 8).

In addition, there was a trend toward an effect of condition on distance traveled *F*_(2,104)_ = 2.907, *p* = 0.059, partial η^2^ = 0.053^co^ (Fig. 8), suggesting neonatal pain might produce a slight hyperactivity. There was no significant effect of condition on number of open arm entries *F*_(2,100)_ = 0.783, *p* = 0.46, partial η^2^ = 0.015^cp^ or on total number of arm entries *F*_(2,101)_ = 1.847, *p* = 0.163, partial η^2^ = 0.035^cq^.

## Discussion

Our data show that repeated acute neonatal pain enhances amygdalar, but not hypothalamic, CRH expression in male, but not female, rats. In addition, we observed a decrease in later anxiety-like behavior that did not appear to be sex specific. Lastly, we observed no long-term differences in maternal rearing behaviors for litters subjected to neonatal manipulations. These data help clarify the mechanisms by which neonatal pain affects later behavior and provide insight into potential targets for future remediation.

Our first key finding is that during the neonatal period, male pups exposed to our acute pain manipulation exhibited higher levels of CRH expression in the amygdala compared to undisturbed (non-handled) controls. Additionally, we found that neonatal pain increases amygdalar CRH and c-fos mRNA colocalization compared to both handled and undisturbed controls. Our findings are the first to report enhanced amygdala CRH expression and enhanced c-fos expression, using a neonatal pain model. Nevertheless, these findings are consistent with previous work demonstrating that repeated instances of cold stress inflicted during the neonatal period increased amygdalar, but not hypothalamic, CRH mRNA expression (Hatalski et al., 1998). Additionally, in the adult literature, chronic painful stimuli has been shown to elevate amygdalar CRH expression (Ulrich-Lai et al., 2006). Although we were unable to find a significant difference in c-fos expression based on condition alone, our mRNA colocalization measure indicates that the amygdala is activated in response to our acute pain model. Adult rodent studies have also indicated that somatic nociception activates c-fos expression in the amygdala (Nakagawa et al., 2003). Thus, our data suggest that the neonatal amygdala may be reacting similarly to the adult amygdala in response to nociceptive stimuli.

This enhanced CRH expression was limited to males and only when compared to the undisturbed controls. Females did not show the same CRH expression pattern as males, in that there was not enhanced CRH or c-fos expression following neonatal pain in the amygdala. These findings are consistent with a prior study using only adult male rodents, which found that the amygdala but not hypothalamic CRH is enhanced following stress (Makino et al., 1999). The current data are also similar to other reports involving early life stress. For example, prenatally-stressed male rats exhibit higher levels of amygdala CRH-R1 mRNA expression in the amygdala at two weeks old (PND13–PND14) compared to both male controls and experimental and control female groups (Brunton et al., 2011). Morphologic sex differences in rodent amygdala size has also been found in neonatally stressed PND20 males (Guadagno et al., 2018) and basal non-stressed adult males (Hines et al., 1992) both of which demonstrated an increase in amygdala size compared to respective female groups. Thus, the currently observed increase in amygdala CRH mRNA in only male rats may be reflective of a broader sex difference in the neurobiology of early-life stress. While the amygdala appears critical in males, other structures, such as the hypothalamus, may serve a similar function in females. For example, females have exhibited higher hypothalamic CRH mRNA expression within the first two weeks of life independent of prenatal stress conditions (Halasz et al., 1997). Additionally, PND14 females, but not males, showed a significant increase in hypothalamic CRH expression after exposure to prenatal stress (Halasz et al., 1997). In the present study, although there were apparent differences in the pattern of CRH mRNA expression in the hypothalamus between male and female subjects, we failed to find a significant effect in the hypothalamus of females which may reflect a lack of power (and therefore a Type II error) rather than a true negative result.

Our second key finding is that neonatal pain decreases anxiety-like behavior in juvenile rats. The EPM is a behavioral assay that has been validated to assess anxiety-like behavior in rodents, relying on a rodent’s inclination toward enclosed spaces and avoidance of open spaces and heights (Walf and Frye, 2007). The current data demonstrate that early life pain increased time spent in open-arms in juvenile subjects. This has been reported several times in the literature. For example, rodents subjected to a single neonatal inflammatory injury displayed increased exploratory behaviors (in the EPM) in adulthood (Anseloni et al., 2005). In addition, non-painful tactile stimulation during the neonatal period exhibited an increase in later-life exploratory behaviors independent of sex in the EPM (Imanaka et al., 2008). Surprisingly, more tonic neonatal manipulations have resulted in no differences in later-life EPM behaviors when compared to both handled and undisturbed controls (Ranger et al., 2018). However, inconsistencies in rodent model (mouse versus rat) and manipulation procedure (4 vs 10 paw pricks daily) between the present study and the previous research likely contribute to these behavioral differences. Finally, acute neonatal pain (but not non-painful handling) manipulations have been shown to reduce later-life fear expression following auditory fear conditioning (Davis et al., 2018). Thus, our findings are most consistent with the literature suggesting that neonatal pain may reduce subsequent fear and anxiety.

Thus, the current data demonstrate that neonatal pain both acutely elevates amygdalar CRH mRNA and causes a subsequent reduction in anxiety. One possible explanation linking these findings is that the high expression of amygdalar CRH during the neonatal period induces a later downregulation of amygdalar CRH signaling. This is consistent with the known physiology of CRH signaling (Dautzenberg and Hauger, 2002). Indeed, exposure to high levels of CRH causing receptor downregulation seems to be a critical component of the HPA-axis negative feedback loop (Aguilera, 1998). Previous work also shows that adult male rodents exhibit a downregulation of amygdalar CRH receptors in response to intracranial administration of CRH (Hauger et al., 1993). However, the developmental effects of high levels of CRH may be more complicated. Intracranial administration of CRH in neonatal rodents identified a subsequent upregulation of hippocampal and hypothalamic, but not amygdalar, CRH1 receptors (Brunson et al., 2002). Thus, the possibility that a compensatory decrease in amygdalar CRH signaling could be a driving force behind our observed juvenile anxiolytic responses needs more investigation.

The observed reduction in later-life anxiety resulting from neonatal adversity could be due, in part, to aspects of our neonatal manipulations that cause resilience-producing changes in maternal behavior, rather than the pain per se. For example, it is well established that dams enhance pup grooming behaviors after litters are exposed to acute neonatal hind paw nociception (Smotherman et al., 1977; Walker et al., 2003) and rodent mothers enhance pup grooming behaviors immediately following 15-min bouts of maternal separation (Walker et al., 2008). These types of changes can mitigate against the effects of stress and trauma (Caldji et al., 1998; Walker et al., 2008; Kentner et al., 2019). Although our study did not find significant differences in maternal behaviors based on litter condition (neonatally manipulated, undisturbed), the current methodology was not designed to assess the acute effects of neonatal pain on maternal behavior, but to investigate whether there were enduring effects of the pain manipulation on dam-pup interactions, a fact which is not well established in the literature. Therefore, measurement occurred before the manipulations began on each day. In this regard, the current data demonstrate that there do not appear to be lasting consequences of neonatal pain on maternal behavior.

Although additional experiments are necessary to further flesh out potential sex differences in the effects of neonatal pain on hypothalamic CRH mRNA expression, the long-term behavioral consequences on other forms of anxiety (such as predator odor exposure), directly link the amygdala CRH system with later behavioral dysfunction, these studies yielded several novel findings. In particular, these data demonstrated that acute neonatal nociception affects early-life amygdala CRH mRNA expression in a sex dependent manner and reduced juvenile innate anxiety.

## Synthesis

Reviewing Editor: Julie Andersen, Buck Institute Decisions are customarily a result of the Reviewing Editor and the peer reviewers coming together and discussing their recommendations until a consensus is reached. When revisions are invited, a fact-based synthesis statement explaining their decision and outlining what is needed to prepare a revision will be listed below. The following reviewer(s) agreed to reveal their identity: Gordon Barr.

Both the reviewers and myself felt that the authors had done a good job responding to previous criticisms. The authors present a much improved manuscript. What is left are a few minor points.

It is recommended in the section titled 'Effects of sex: CRH and c-fos Expression and Colocalization' on p.16-17, the authors should make clear that levels of CRH mRNA expression do not necessarily reflect the actual amount of active CRH peptide being secreted from those cells. Neuropeptides are not active until they are released from a cell, and they undergo complex biogenesis, transport, and secretion from vesicles which is often independent of mRNA levels.

The photomicrographs are far too low power to see any detail at all and should have a least a couple of high power examples.

Text:

Line 315 states 6 days but days 2-6 are only 5.

Lines 422, 423. In the discussion please acknowledge that the difference in the amygdala CRH mRNA between was between non-handled and pain only. The importance of this mitigated by the colocalization data, but is worth noting.

Line 552 and later.. Make it clear that CRH and c-fos colocalization was of the message not protein.

Figures.

Figures 6 & 7 need to be redone. It needs labels for the columns of micrographs. It is also impossible to see any detail in them. We need at least one high power panel to see the individual transcripts and the colocalization. Figure 3 would also benefit from one or more higher power micrographs as again it is impossible to see any details.
